# An Improved Caulobacter crescentus Operon Annotation Based on Transcriptome Data

**DOI:** 10.1128/MRA.01025-20

**Published:** 2020-10-29

**Authors:** Mohammed-Husain Bharmal, James R. Aretakis, Jared M. Schrader

**Affiliations:** a Department of Biological Sciences, Wayne State University, Detroit, Michigan, USA; Indiana University, Bloomington

## Abstract

Caulobacter crescentus is a model alphaproteobacterium with a well-studied genetic network controlling its cell cycle. Essential for such studies is an accurate map of the expressed features of its genome. Here, we provide an updated map of the expressed RNAs by integrative analysis of 5′ global rapid amplification of cDNA ends, transcriptome sequencing, rifampicin treatment RNA sequencing, and RNA end-enriched sequencing data sets.

## ANNOUNCEMENT

The operon map presented here is based on the latest Caulobacter crescentus GenBank entry (GenBank accession number NC_011916.1) (1, 2), with the latest operon and transcriptional unit (TU) definitions (2). The original mRNA operon annotation was downloaded from reference 3. To include noncoding RNAs (ncRNAs) in operons, ncRNAs that were within 50 nucleotides (nt) of another ncRNA or operon were combined into a single operon. Due to the recent availability of multiple types of transcriptome sequencing (RNA-seq) data, we downloaded 5′ RNA end data to denote transcription start sites (TSSs) (5′ global rapid amplification of cDNA ends [RACE] [3] and RNA end-enriched sequencing [Rend-seq] [4] data), RNA density data (RNA-seq [2] and Rend-seq [4] data), and 3′ RNA end data (Rend-seq [4] data). For each operon in the genome, we scanned for the 5′-most PPP-enriched RNA ends from the 5′ global RACE data, which were labeled TSSs. If no TSS was found for a particular operon, then Rend-seq data were used to search for a probable TSS. Because Rend-seq cannot distinguish between 5′-PPP ends generated by transcription and 5′-P ends generated by RNA processing, only the 5′-most Rend-seq end was reported for a given operon as the operon’s probable TSS. We scanned for TSSs using a 300-nt window from the operon’s first gene or, for RNA operons such as rRNA, we searched 300 nt upstream of the processed 5′ ends; 300 nt was chosen since this was approximately the size of the largest 5′ untranslated region in the C. crescentus genome (2). For each 300-nt upstream region, the farthest upstream TSS was labeled P1, and each subsequent TSS from 5′ to 3′ was annotated sequentially. Since Rend-seq data showed that limited numbers of operons had well-defined 3′ ends, we did not refine the 3′ ends of operons.

Because RNA processing can lead to the generation of stable functional RNAs (5, 6), we also utilized rifampicin treatment RNA sequencing (RIF-Seq) data (NCBI GEO accession number GSE157432) to map the coordinates of stable processed RNAs. We used RIF-seq data from 15 min after rifampicin treatment, scanned along the length of the genome, and annotated stable RNAs that were longer than 20 nt, for which each nucleotide was above 30 reads, and as a whole averaged above 43 reads/nucleotide. The average read cutoff value was determined empirically using tRNAs since their biogenesis requires both 5′ and 3′ processing. Rend-seq-derived 5′ ends were not utilized for this analysis since they could arise from RNA decay intermediates. Since RNA-seq coverage can be noisy, if multiple stable RNAs were identified within 20 nt and the RNA-seq reads remained above zero between them, then the regions were manually combined into a single stable RNA feature.

**FIG 1:**
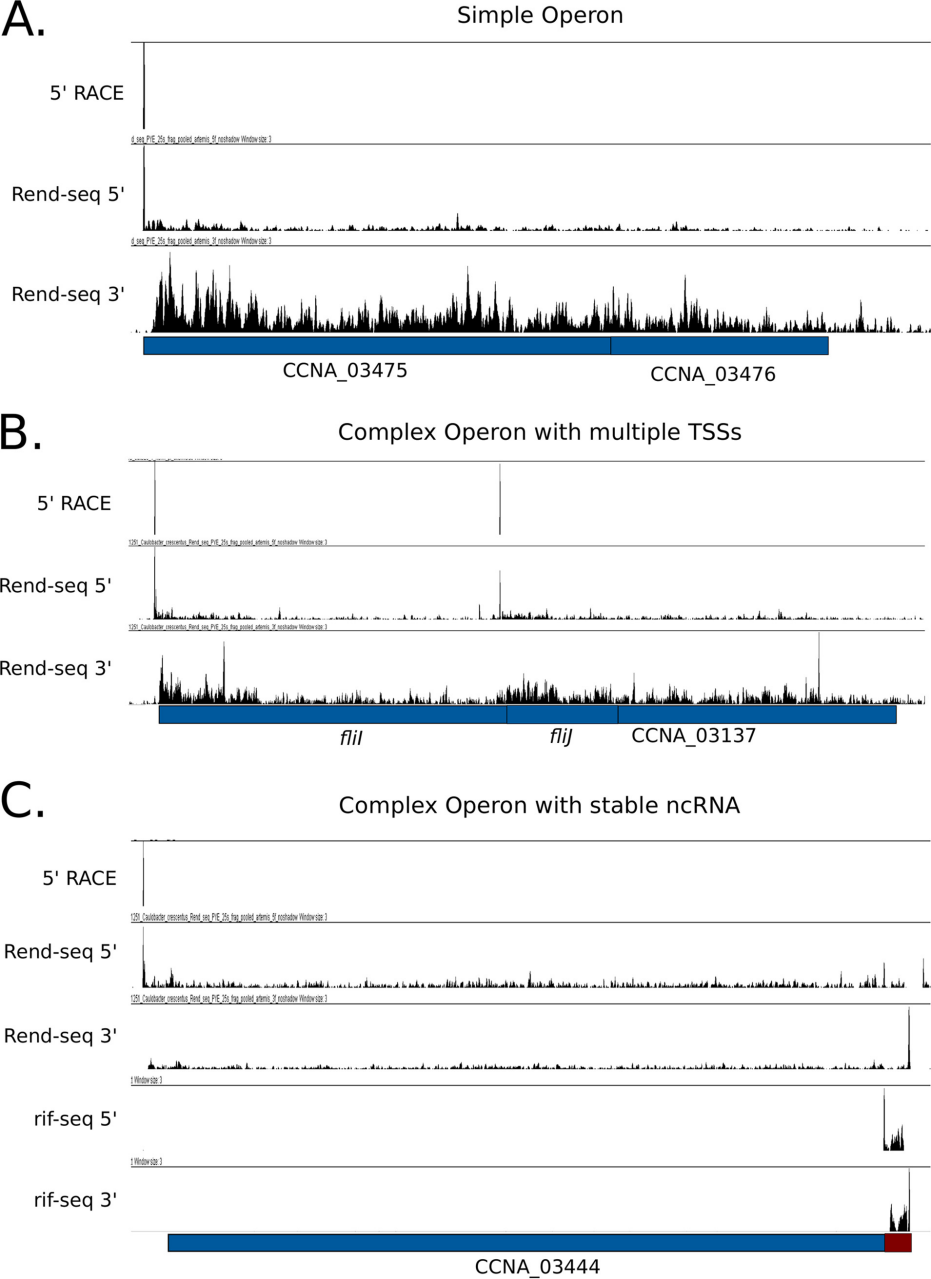
Examples of simple and complex operons. (A) A simple operon containing one predicted TU. (B) A complex operon containing more than one TSS driving multiple TUs. (C) A complex operon with a single predicted TU harboring a stable ncRNA.

Finally, we classified operons as simple if the RNA was made from a single TU defined by a single TSS and lacked any observable RNA processing sites (Fig. 1A). Operons were classified as complex if they were defined by multiple TUs (arising from multiple upstream TSSs or the presence of internal TSSs) (Fig. 1B) or if they contained stable processed RNAs within the operon coordinates (Fig. 1C and Table 1).

**TABLE 1:** Summary of C. crescentus operons

Operon type	No.
Total operons	2,915
Operons with primary TSS mapped	2,205
Operons with TSS(s)	1,790
Operons with only probable TSS	415
Operons with no primary TSS mapped	710
Operons updated in this resource	517
Simple operons	1,635
Complex operons	692
Operons with multiple primary TSSs	220
Operons with stable processed ncRNAs	76
Operons with internal primary TSSs	460
Operons that were not determined to be simple or complex	588

In total, 517/2,914 operon annotations were updated upon this analysis (Table 1). At least one primary TSS was identified for 2,205 of the 2,915 predicted operons, while no TSS was identified for 710. The lack of TSS identification is likely due to the RNA data sets being collected under only two growth conditions (5′ global RACE data were collected from cells grown in M2 minimal salt medium with 0.2% glucose, and Rend-seq data were collected only from cells grown in peptone-yeast extract medium). A total of 692 complex operons containing multiple RNA isoforms generated from the same DNA locus, which can potentially have different regulation and functions, were identified. Since some complex C. crescentus operons, such as the *ctrA* operon, have multiple regulatory inputs that help to coordinately regulate the expression of this essential cell cycle master regulator (7), we think that the updated operon map provides a useful tool for the interpretation of *Caulobacter* gene expression data.

### Data availability.

The TU map described is accessible at schraderlab.org/resources and https://doi.org/10.6084/m9.figshare.12919208. A GenBank file of the results and browsable versions of the raw data sets are also included in the figshare repository. The RNA-seq data sets are available from the NCBI GEO database with the accession numbers GSE57365 (5′ global RACE data), GSE95211 (Rend-seq data), GSE54883 (RNA-seq data), and GSE157432 (RIF-seq data).
